# Endoscopic submucosal dissection as a treatment for gastric subepithelial tumors that originate from the muscularis propria layer: a preliminary analysis of appropriate indications

**DOI:** 10.1007/s00464-013-2904-9

**Published:** 2013-03-22

**Authors:** Seung Yeon Chun, Kyoung Oh Kim, Dong Seon Park, In Joung Lee, Ji Won Park, Sung-Hoon Moon, Il Hyun Baek, Jong Hyeok Kim, Choong Kee Park, Mi Jung Kwon

**Affiliations:** 1 Division of Gastroenterology and Hepatology, Department of Internal Medicine, Hallym University Sacred Heart Hospital, Hallym University, College of Medicine, 896, Pyeongchon-dong, Dongan-gu, Anyang, Gyeonggi-do 431-070 Korea; 2 Department of Pathology, Hallym University Sacred Heart Hospital, Hallym University, College of Medicine, Anyang, Korea; 3 Department of Internal Medicine, Gachon University Gil Medical Center, Incheon, Korea

**Keywords:** Subepithelial tumor, Muscularis propria, Endoscopic submucosal dissection

## Abstract

**Background:**

Endoscopic submucosal dissection (ESD) is a well-established method for the treatment of gastrointestinal epithelial tumors. However, the treatment of gastric subepithelial tumors (SETs) that originate from the muscularis propria layer still depends primarily on surgical techniques. We evaluated the appropriate indications for ESD in the treatment of SETs that originate from the muscularis propria layer.

**Methods:**

Thirty-five patients with gastric SETs that originate from the muscularis propria layer who underwent ESD were enrolled, and the charts were retrospectively reviewed to investigate the parameters predictive complete resection and complications.

**Results:**

The mean age of the patients was 54.15 ± 9.3 years, and the male/female ratio was 2:3. Twenty-eight of the 35 SETs (85.7 %) were movable, and 15 (45.7 %) had a positive rolling sign. The most frequent location of the SETs was high body (*n* = 14). The most common pathological diagnoses were leiomyoma (60 %) and gastrointestinal stromal tumor (28.6 %). The complete resection rate was 74.3 %. A positive rolling sign (*p* = 0.022) and small tumor size (≤20 mm; *p* = 0.038) were significantly associated with complete resection. Two patients (6.1 %) developed perforations that required surgical treatment; their SMTs were neurogenic tumors with fixed lesion. Tumor mobility was significantly associated with perforation (*p* = 0.017).

**Conclusions:**

The ESD method appears to be relatively safe for use in the complete resection of SETs that originate from the muscularis propria layer. Small tumor size (≤20 mm) and a positive rolling sign are appropriate indications for ESD.

Gastric subepithelial tumors (SETs) are common lesions in the upper gastrointestinal tract and often are found incidentally during endoscopic examination [1]. SETs often are considered to be relatively benign; however, they have malignant potential, particularly if they originate from the muscularis propria layer [2]. Gastrointestinal stromal tumor (GIST), the most common neoplasm that originates from the muscularis propria layer of the gastrointestinal tract, is diagnosed as malignant in 10–30 % of cases. The nature of GISTs cannot be determined based solely on endoscopic imaging [3, 4]. Endoscopic ultrasound (EUS) is the most accurate method for differentiating gastrointestinal SETs; however, in most cases, it is not sufficient for making a definite diagnosis of hypoechoic lesions in the third or fourth echo layer [5, 6]. An accurate pathological confirmation therefore is necessary for differentiating between benign and malignant SETs.

Many tissue sampling techniques have been developed for the definitive diagnosis of SETs, including bite-on-bite biopsy, EUS-guided fine needle aspiration (EUS-FNA), and EUS-guided core needle biopsy [2, 4]. However, these techniques are not always sufficient for diagnosis, and normal pathological reports from EUS-FNA or EUS-guided core needle biopsy cannot rule out the possibility of malignancy. More aggressive techniques for tissue diagnosis, such as tumor biopsy following incision or unroofing (partial removal of the overlying mucosa) and endoscopic submucosal-mucosal resection (ESMR), have a better diagnostic yield; however, the available data on the effectiveness of these techniques are limited [7–9].

Endoscopic submucosal dissection (ESD) has been developed in recent years as a technique for the treatment of early gastric cancer [10]. The ESD technique allows precise en bloc resection regardless of the size and shape of the lesion; however, evidence regarding the efficacy and safety of ESD in the resection of SETs is limited. An initial study suggested that complete en bloc resection using ESD is indicated only for SETs that originate from the muscularis mucosa or submucosal layer [8]. ESD for the resection of SETs that arise from the muscularis propria layer has been recently introduced in several feasibility studies and has shown a complete resection rate of approximately 64–75 % [11–13]. However, ESD appears to be associated with technical difficulty and a higher incidence of complications, such as perforation and bleeding, and the treatment of SETs that originate from the muscularis propria layer still depends primarily on surgical techniques.

In this study, we evaluated the appropriate indications for ESD in SETs that originate from the muscularis propria layer. We retrospectively reviewed data from patients who underwent ESD for gastric SETs to determine the predictors of complete resection and the possible complications of ESD.

## Materials and methods

### Patients and study design

Between March 2009 and May 2012, 35 ESD procedures were performed in consecutive patients at the Hallym University Sacred Heart Hospital, Anyang, Korea, who had gastric SETs that originated from the muscularis propria layer. Patients were considered eligible for ESD if the tumor originated from the muscularis propria layer and did not show extraluminal growth as confirmed by EUS. The data were collected retrospectively, and the charts were reviewed for the following information: clinical characteristics, endoscopic and EUS findings, and clinical outcomes.

All of the patients included in the study provided written, informed consent to undergo ESD following detailed verbal and written explanations of the ESD procedure and other possible treatment options. This study was approved by the institutional review board of the hospital.

### Standard endoscopy and EUS

All of the patients underwent a routine upper GI endoscopy (GIF-Q260; Olympus Optical Co., Tokyo, Japan). The assessment of each tumor included the location, size, appearance, extent, mobility, and consistency of the lesion. The location of the lesion was classified based on six sections: cardia, fundus, high body, midbody, lower body, and antrum. When the tumor was freely movable more than half of maximal tumor diameter upon palpation with biopsy forceps, the lesion was considered to have a positive rolling sign.

**Fig. 1:**
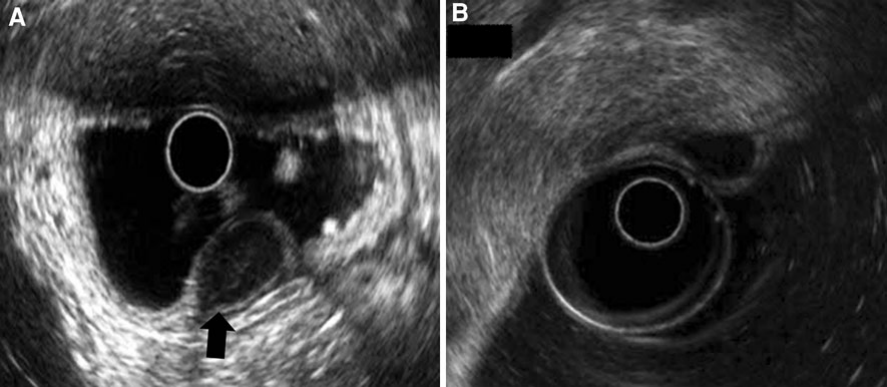
The degree of muscular connection with a subepithelial tumor as shown by endoscopic ultrasound. **A** Narrow muscular connection with the tumor: the diameter of the connection with the fourth layer of the tumor is <50 % of the maximal diameter of the tumor base (*black arrow* hyperechoic demarcation line between the tumor and the muscularis propria layer). **B** Wide muscular connection with the tumor: the diameter of the connection with the fourth layer is >50 % of the maximal diameter of the tumor base

EUS was performed with a radial-scanning echo endoscope (Olympus GF-UM2000, 6 MHz and 12 MHz; Olympus Optical Co.) to assess the layer of origin. Other features that were identified by EUS included the growth pattern (intragastric vs. extragastric), the demarcation of margin, and the degree of the connection area (narrow vs. wide) with the fourth layer of the tumor. A narrow versus wide muscular connection was defined as a connection area with the fourth layer that was ≤50 versus >50 % of the maximal diameter of the tumor base, respectively (Fig. 1) [11].

### ESD procedures

Before the ESD procedure, each patient was sedated using an intravenous propofol infusion to produce a deep sedation level and his/her heart rate, blood pressure, and oxygen saturation were closely monitored. The ESD procedure was performed using a video endoscope (GIF-Q260J; Olympus Optical Co.) with a hood (length, 4 mm) attached at the endoscope tip. After the target lesion was identified, marking dots were placed circumferentially at its margin using an argon plasma coagulation (APC) probe under an electrosurgical coagulation current (forced coagulation, 40 W). A mucosal incision was made with a needle-knife (KD-10Q-1L; Olympus Optical Co.) along the line of dots following the injection of a mixture of glycerol (10 %), epinephrine (dilution 1:100,000), and a small amount of indigo carmine. An insulated-tip knife (KD-611; Olympus Optical Co.) was inserted into the initial incision, and an electrosurgical current was applied using an electrosurgical generator (Erbe VIO 300D; Tübingen, Germany) in EndoCut mode (effect 2) to make an incision around the lesion.

After the circumferential incision of the overlying mucosa, the tumor was exposed below the submucosal layer. The insulated-tip knife (coagulation mode, effect 5, 60 W) was used in combination with additional saline and indigo carmine injections to separate carefully the tumor from the muscularis propria layer. The procedure time, defined as the required time from the marking to the resection of the lesion, was recorded for each procedure. The resection wound was observed carefully to evaluate whether any residual tumor was present.

### Pathological examination and follow-up assessment

**Fig. 2:**
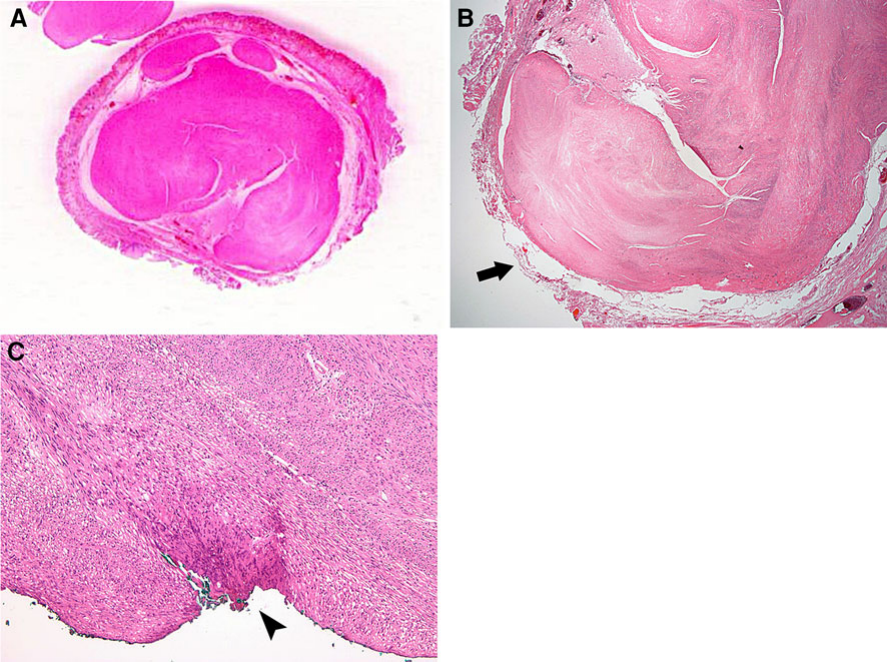
**A** Magnified scan of an H&E slide showing a well-demarcated submucosal tumor with clear circumferential resection margins following endoscopic submucosal dissection. **B** The deep resection margin (*arrow*) is negative (R0) (×40). **C** The resection margin, indicated by *green color* and an *arrowhead*, is involved with a gastrointestinal stromal tumor (R1) (×200)

The tissue specimens were fixed in formalin solution. The histological evaluation included the identification of cell type, overall cellularity, nuclear atypia, immunohistochemical findings, and the mitotic index. Immunohistochemical analyses of c-kit (CD 117), CD 34, smooth muscle actin (SMA), and S-100 markers were performed to identify the tumor subtype. The resection margin was examined microscopically, and its status was determined on the basis of the general criteria of cancer involvement in resection margin [14]. The completeness of resection was classified according to the extension of tumor cells into the resection margin: (1) complete (R0) resection, in which the lateral and vertical resection margins were free of tumor; (2) microscopic incomplete (R1) resection, in which the tumor extended into the lateral or vertical resection margin; and (3) macroscopic incomplete (R2) resection, in which the tumor could not be completely resected according to its endoscopic aspects (Fig. 2).

Each patient who underwent ESD was followed up by standard endoscopy within the 2-month period following the procedure to confirm the healing of the artificial ulcer and to rule out local recurrence. The patients who had GISTs underwent surveillance examinations every 6 months, and surveillance studies included both endoscopy and computed tomography of abdomen and pelvis.

### Outcome measurements

The major outcome measurements were complete resection rate, procedure-related complication rate, and diagnostic parameters predictive of complete resection. The complete resection rate was defined as the proportion of tumors that were removed en bloc by R0 resection. Procedure-related complications included perforation and bleeding. Perforation was diagnosed when mesenteric fat or the intra-abdominal space was directly observed during the procedure (frank perforation) or when free air was observed on a plain chest X-ray following the procedure. If an endoscopically visible perforation was observed during the procedure, it was closed with hemoclips and additional surgical management was recommended. Major bleeding was defined as continued bleeding that required the administration of ≥2 U of packed RBCs (PRBCs) or uncontrolled bleeding that persisted despite endoscopic intervention with at least two hemostatic modalities.

### Statistical analyses

The *χ* ^2^ test or Fisher’s exact test was used for comparisons of categorical data, and the *t* test was used for the analysis of continuous data. A *p* value of <0.05 was considered to be statistically significant. All of the data were analyzed using the SPSS software program (version 16.0 for Windows; SPSS Korea, Seoul, Korea).

## Results

### Characteristics of the patients and the tumors

A total of 35 adult patients with gastric SETs underwent ESD at Hallym University Sacred Heart Hospital. The characteristics of these patients are summarized in Table 1. The mean age of the patients was 54.15 ± 9.3 years (range 31–71 years), and the male/female ratio was 2:3. The mean follow-up duration of the study subjects was 6.13 ± 9.02 months (range 1–44.3 months).

**Table 1:** Characteristics of the patients and the tumors (*n* = 35)

Sex (female/male)	14/21
Age (year; mean ± SD)	54.15 ± 9.3
Follow-up duration (month; mean ± SD)	6.13 ± 9.02
Endoscopic findings	
Rolling sign, *n* (%)	
Positive	16 (45.7 %)
Negative	19 (54.3 %)
Mobility, *n* (%)	
Fixed	5 (14.3 %)
Mobile	30 (85.7 %)
Location, *n* (%)	
Fundus	1 (2.9 %)
Cardia	10 (28.6 %)
High body	14 (40 %)
Mid body	3 (8.6 %)
Lower body	4 (11.4 %)
Antrum	3 (8.6 %)
Endoscopic ultrasound findings	
Connection with the fourth layer on EUS, *n* (%)	
Narrow	12 (34.3 %)
Wide	23 (65.7 %)
Margin of tumor	
Well-demarcated	34 (97.1 %)
Not well-demarcated	1 (2.9 %)
Pathological diagnosis	
Leiomyoma	21 (60 %)
GIST	10 (28.6 %)
Neurogenic tumor	2 (5.7 %)
Other	2 (5.7 %)
Tumor diameter (mm; mean ± SD)	17.99 ± 7.86

Standard upper GI endoscopy revealed that 30 of the 35 SETs (85.7 %) were movable by biopsy forceps and that 15 SETs (45.7 %) of these mobile tumors had a positive rolling sign. The most frequent location of the SETs was high body (*n* = 14, 40 %), followed by cardia (*n* = 10, 28.6 %), lower body (*n* = 4, 11.4 %), mid body (*n* = 3, 8.6 %), antrum (*n* = 3, 8.6 %), and fundus (*n* = 1, 2.9 %). EUS revealed a narrow connection with the fourth layer in 12 tumors (34.3 %) and a well-demarcated margin in 34 tumors (97.1 %).

Twenty-one patients (60 %) had leiomyomas, and ten patients (28.6 %) had GISTs. Other histological diagnoses included neurogenic tumor (*n* = 2), gastritis cystica profunda (*n* = 1), and inflammatory fibroid polyp (*n* = 1). The mean maximal diameter of the tumors was 17.99 ± 7.86 mm (range 8–40 mm). The HPF mitotic counts of most resected tumors were low (<5 mitotic figures/50 HPF), suggesting a low risk of malignancy. Two SETs were found to have a greater than moderate risk of malignancy. One patient had a GIST of moderate risk (mitotic figures, 9/50 HPF; size, 2.2 cm) with an R1 resection, but she refused additional surgical management. The other patient had a GIST with high risk (mitotic figures, 15/50 HPF; size, 2.2 cm), and an R1 resection was performed. This patient underwent a gastric wedge resection and adjuvant chemotherapy with imatinib mesylate (Gleevec; Novartis Pharmaceuticals, Basel, Switzerland) for 1 year. No recurrence has been detected to date during follow-up (28 months).

### Clinical outcomes

#### Resection rate

A successful complete resection (R0) by ESD was achieved in 26 of 35 tumors (success rate, 74.3 %). We analyzed the rate of complete resection according to tumor size, routine endoscopic and EUS features, and histological diagnosis (Table 2). The complete resection rate was significantly different in cases of a tumor size ≤20 mm than in cases of a tumor size >20 mm (87 vs. 50 %, respectively; *p* = 0.038). A positive rolling sign was significantly associated with more frequent complete resection (93.8 vs. 57.9 %, *p* = 0.022). The other parameters determined by EUS (demarcation of the margin and the degree of connection with the fourth echo layer) did not show a correlation with the complete resection rate.

**Table 2:** Predictive parameters for complete resection in patients who underwent endoscopic submucosal dissection for gastric subepithelial tumors

Parameters	Complete resection		*p* value
	Yes (*n* = 26)	No (*n* = 9)	
Rolling sign, *n* (%)			**0.022**
Yes	15 (93.8 %)	1 (6.3 %)	
No	11 (57.9 %)	8 (42.1 %)	
Mobility, *n* (%)			0.095
Fixed lesion	2 (40 %)	3 (60 %)	
Mobile lesion	24 (80 %)	6 (20 %)	
Margin of tumor, *n* (%)			0.257
Well-demarcated margin	26 (76.5 %)	8 (23.5 %)	
No well-demarcated margin	0 (0 %)	1 (100 %)	
Connection with the fourth layer on EUS, *n* (%)			0.121
Narrow connection	11 (91.7 %)	1 (8.3 %)	
Wide connection	15 (65.2 %)	8 (34.8 %)	
Tumor size, *n* (%)			**0.038**
≤20 mm	20 (87 %)	3 (13 %)	
>20 mm	6 (50 %)	6 (50 %)	
Histological diagnosis, *n* (%)			0.291
Leiomyoma	18 (85.7 %)	3 (14.3 %)	
GIST	6 (60 %)	4 (40 %)	
Neurogenic tumor	1 (50 %)	1 (50 %)	
Other	1 (50 %)	1 (50 %)	

Bold values indicate statistically significant parameter

#### Complications

The mean ESD procedure time was 32.29 ± 20.55 min (range 7–84 min). No immediate postprocedure complications were observed. Two patients (6.1 %) developed perforations that required surgical treatment; no patient developed major bleeding (Table 3). In one of the cases of perforation, the tumor was tightly adherent to the muscularis propria layer, and an endoscopically visible perforation was observed during the procedure. An emergent operation was performed, and the final pathological diagnosis of the tumor was neurilemoma. In the other case of perforation, a visible perforation was observed after removal of the tumor. The patient underwent a wedge resection, and the pathological diagnosis of the tumor was schwannoma with a clear resection margin.

**Table 3:** Clinical outcomes of endoscopic submucosal dissection for the study subjects (*n* = 35)

Resection	*n* (%)
R0	26 (74.3 %)
R1	6 (17.1 %)
R2	3 (8.6 %)
Procedure time (min; mean ± SD)	32.29 ± 20.55
Major bleeding, *n* (%)	0 (0 %)
Perforation, *n* (%)	2 (5.7 %)

The predictive parameters of perforation are shown in Table 4. A positive rolling sign, small tumor size (<20 mm), and wide connection with the fourth echo layer on EUS were not significantly associated with perforation. Both cases of perforation involved fixed lesions, and tumor mobility was significantly associated with perforation (*p* = 0.017).

**Table 4:** Predictive parameters of perforation in patients who underwent endoscopic submucosal dissection for gastric subepithelial tumors

Parameters	Perforation		*p* value
	Yes (*n* = 2)	No (*n* = 33)	
Rolling sign, *n* (%)			0.489
Positive	0 (0 %)	16 (100 %)	
Negative	2 (10.5 %)	17 (89.5 %)	
Mobility, *n* (%)			**0.017**
Fixed lesion	2 (40.0 %)	3 (60.0 %)	
Mobile lesion	0 (0 %)	30 (100 %)	
Connection with fourth layer on EUS, *n* (%)			0.536
Narrow connection	0 (0 %)	12 (100 %)	
Wide connection	2 (8.7 %)	21 (91.3 %)	
Tumor size, *n* (%)			0.999
≤20 mm	1 (4.3 %)	22 (95.7 %)	
>20 mm	1 (8.3 %)	11 (91.7 %)	
Histological diagnosis, *n* (%)			**0.002**
Leiomyoma	0	21	
GIST	0	10	
Neurogenic tumor	2	0	
Other	0	2	

Bold values indicate statistically significant parameter

## Discussion

The clinical application of ESD, including its application in the treatment of SETs that originate from the muscularis propria layer, has increased in recent years [11–13]. However, this technique is still in an experimental phase, and no studies have addressed its safety, long-term outcomes, or appropriate indications. Most studies to date on the endoscopic resection of SETs have been conducted on a small scale and have been concerned only with technical feasibility. However, a few recent studies have investigated the factors that are related to complete resection and to complications.

GISTs are the most common type of SET and are the primary target of treatment. GISTs are potentially malignant and have a high postoperative recurrence rate. It therefore is preferable that the purpose of treatment should be microscopic complete resection (R0 resection) with a microscopically negative margin rather than macroscopic complete resection. Because macroscopic complete resection has been considered to be equivalent to complete resection in most previous reports on the endoscopic resection of SET, the assessment of the usefulness of endoscopic resection has been limited.

**Fig. 3:**
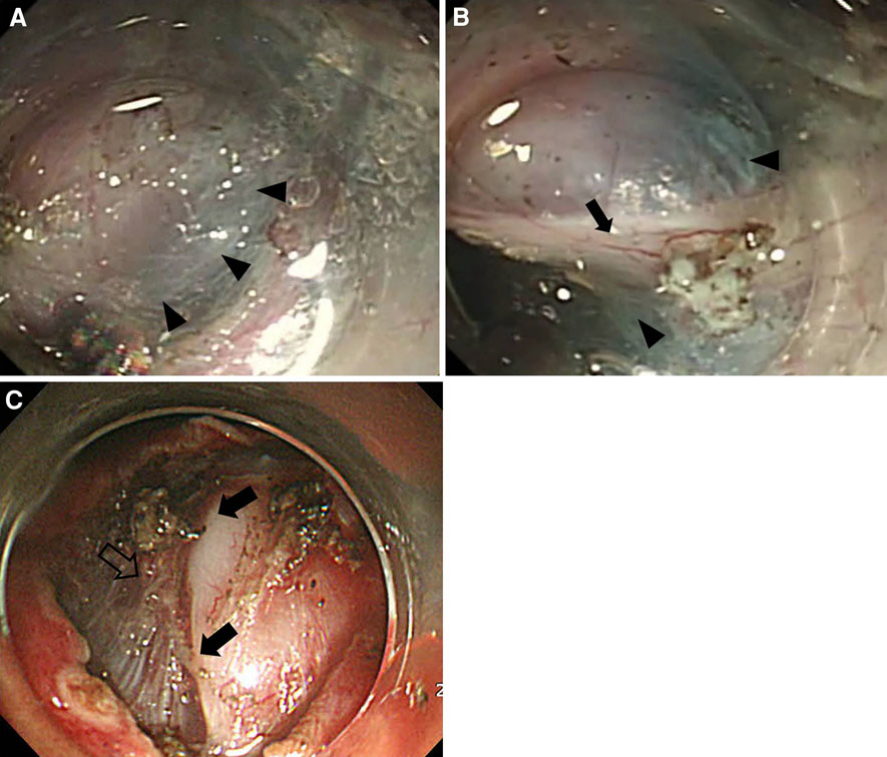
Endoscopic findings obtained from the endoscopic submucosal dissection of a gastric subepithelial tumor. **A** The tumor base was surrounded by a bluish submucosal layer (*black arrowheads*) following the injection of a mixture of glycerol and indigo carmine. **B** Following the dissection of the submucosal layer, a whitish narrow muscular connection area (*black arrow*) was observed. **C** Wide muscular connection area (*black arrows*) and a muscularis propria layer (*empty arrow*) adjacent to the tumor were exposed following a circumferential incision around the lesion

Białek et al. [11] recently analyzed factors related to the rate of endoscopic complete resection (R0 resection) and to complications following ESD for SET. These authors reported that the area connected to the muscularis propria layer was a factor related to complete resection, whereas the tumor size and location were not. They performed EUS to assess the connection area between the tumor and the muscularis propria layer. In the present study, a positive rolling sign and tumor size ≤2 cm were found to be factors related to complete resection. A positive rolling sign indicates that the tumor originated from the submucosal layer or has a very narrow connection (or no connection) to the muscularis propria layer. In the present study, all tumors that had a positive rolling sign were SETs that originated from the muscularis propria layer and had a narrow muscular connection. When the hyperechoic line between the tumor and the muscularis propria layer was found to be ≥50 % of the maximal diameter of the tumor base by EUS, a narrow muscular connection was confirmed. Because the hyperechoic line was either not observed or was found to be <50 % of the maximal diameter of the tumor base in some cases, it was not possible to consistently identify a narrow muscular connection by EUS alone. Białek et al. reported that EUS was only 73 % accurate in determining the layer of origin of tumors. In the present study, in the cases of tumors that had a narrow muscular connection, a significant portion of the tumor base was loosely connected to the submucosal tissue (Fig. 3A, B). A higher R0 resection rate therefore was expected because of the relative ease of performing a dissection without tumor surface injury. In the cases of tumors that had a wide muscular connection, a substantial portion of the tumor base was tightly connected to the muscle layer (Fig. 3C). A greater risk of perforation or incomplete resection (R1 or R2) therefore was expected because dissection is performed on a large area of the muscularis propria layer.

Białek et al. reported a 68 % R0 resection rate for tumors that originated from the muscularis propria layer, whereas Li et al. reported a 94 % R0 resection rate. The overall R0 resection rate in the present study was 74.3 %. The R0 resection rate for tumors with a positive rolling sign (93.8 %) was significantly higher than that for tumors with a negative rolling sign (57.9 %). Białek et al. reported that tumor size was not related to the complete resection rate. In the present study, the R0 resection rate for tumors ≤2 cm in size (85 %) was significantly higher than that for tumors >2 cm in size (50 %). In theory, as the dissection area increases in proportion to the tumor size, the possibility of incomplete resection also increases. Further studies involving more cases are needed to test this hypothesis.

Complications related to treatment, both of which were instances of perforation, occurred in two cases. Fixed tumor mobility and the presence of a histologically diagnosed neurogenic tumor appeared to be factors related to the occurrence of perforation.

**Fig. 4:**
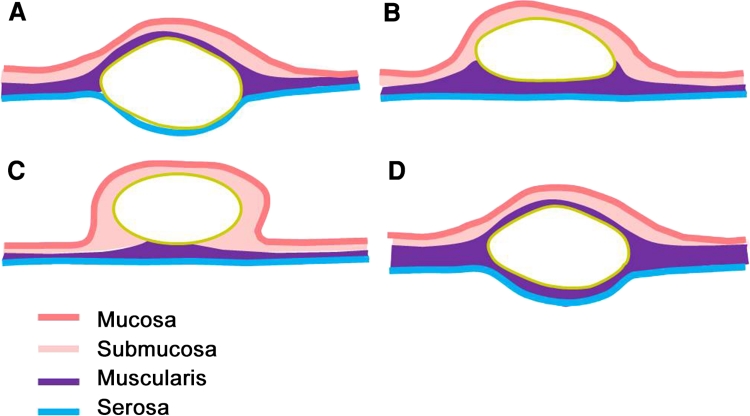
Schematic illustrations of subepithelial tumors with differing growing patterns and muscular connections. **A** Subserosal. **B** Submucosal with a wide muscular connection. **C** Submucosal with a narrow muscular connection. **D** Intramural type

When a tumor was found to be fixed or immobile upon palpation with biopsy forceps, a broad muscular connection was shown by EUS. High tumor mobility is expected in the case of a submucosal growing tumor with a narrow muscular connection. Lower tumor mobility is expected in the case of a submucosal growing tumor with a broad muscular connection or in the case of an intramural-type or subserosal growing tumor (Fig. 4). A subserosal growing tumor shows an extraluminal bulging pattern on EUS and is easily diagnosed. In contrast, an intramural-type tumor is difficult to diagnose by EUS, and the accuracy of EUS diagnosis in terms of the degree of muscular connection is unsatisfactory. The measurement of tumor mobility and the detection of a positive rolling sign, in addition to EUS findings, therefore will be helpful in predicting the success rate of endoscopic resection or the occurrence of complications.

In the present study, perforation occurred in cases of schwannoma and neurilemoma, both of which are neurogenic tumors. This finding is consistent with findings reported in other studies [15]. In cases of gastric schwannoma, the intramural type has been reported to be the most common. It is difficult to dissect intramural-type schwannoma from the adjacent muscle tissue because, in contrast to soft tissue schwannoma, there is no encapsulation and perforation therefore occurs frequently [16, 17].

In cases of intermediate or small SETs that originate from the muscularis propria layer, it is difficult to choose an optimal treatment strategy because preoperative diagnosis and the assessment of the malignant potential of these tumors also are difficult. Endoscopic resection of SETs is less invasive than surgical resection and has high diagnostic accuracy. However, endoscopic resection is not widely used because of its disadvantages, particularly the high risk of incomplete resection and of complications, including perforation. The present study was conducted to analyze factors related to the complete resection rate of endoscopic resection and to the occurrence of complications and to investigate the appropriate indications and contraindications of endoscopic resection. We found that tumors ≤2 cm in size or with a positive rolling sign showed high complete resection rates and almost no complications. Such tumors are therefore presumed to be ideal candidates for endoscopic resection. Endoscopic resection should not be considered in cases of neurogenic tumors or tumors that have no mobility.

The present study has some limitations. First, in view of the small number of patients studied, it is difficult to generalize our results. Although GIST is known as the most common SET, leiomyoma is more common than GIST in our results. Because endoscopically resected SETs were relatively small in size, we could not exclude the possibility of selection bias. Second, a long-term follow-up study has not yet been completed. In cases of GIST in particular, it is desirable to establish a management strategy following endoscopic resection based on the results of a long-term follow-up study. For the patients who underwent endoscopic resection, a regular follow-up study is underway, and the results of a long-term follow-up study involving the GIST patients in particular will be available later. The present study suggests that endoscopic resection (i.e., ESD) may be a good alternative to surgical resection for the treatment of SETs, including GISTs, that originate from the muscularis propria layer. Although this study provides a preliminary analysis of the appropriate indications for ESD, further studies are needed to confirm or extend our findings.
